# Type I Lip Pattern in at Least One Quadrant among First and Second-year Medical and Dental Students of a Medical College: A Descriptive Cross-sectional Study

**DOI:** 10.31729/jnma.6324

**Published:** 2021-04-30

**Authors:** Sarbada Makaju, Sonam Chaudhary, Chandra Kala Rai

**Affiliations:** 1 Department of Anatomy, Kathmandu Medical College and Teaching Hospital, Duwakot, Bhaktapur, Nepal; 2 Department of Physiology, Institute of Medicine, Mahargunj, Kathmandu, Nepal; 3 Department of Physiology, Kathmandu Medical College and Teaching Hospital, Duwakot, Bhaktapur, Nepal

**Keywords:** *grooves*, *identification*, *lips*, *patterns*

## Abstract

**Introduction:**

The upper and lower lip consists of the numerous amounts of fine grooves. This pattern of grooves will be definite throughout the lifetime. The groove of the lip shows variations and play important role in forensic medicine and crime investigation. The objective of this study is to find out the prevalence of type I lip print in at least one lip quadrant among first- and second-year medical and dental students of a medical college.

**Methods:**

The study was conducted on 240 students of a medical college between November 2020-January 2021 after getting the ethical clearance from the Institutional Review Committee (reference no. KMC-IRC 0311202006). The convenient sampling was done. The patterns of the grooves of the lip were studied. The data was analysed with Statistical Package for the Social Sciences 20 version. Point estimate at 95% Confidence Interval was calculated along with frequency and percentage for binary data.

**Results:**

Out of 240 students, type I lip pattern was seen in at least one quadrant in 190 (79.6%) (73.38–84.94 at 95% CI) students. One hundred and fifty-nine (66.3%) had type I pattern in the right upper quadrant, 160 (66.7%) in left upper quadrant, 181 (75.4%) in right lower quadrant, and 177 (73.8%) in left lower quadrant. Type Ia was maximum found in 115 (47.9%) and type Ib in 66 (27.5%) in lower left quadrant of lip.

**Conclusions:**

Our findings showed a higher prevalence of type I lip pattern than those reported by other national studies done among medical students.

## INTRODUCTION

The lips are considered as an essential aspect of the human face. The lips functions are mastication, phonation, facial expression, physical attraction and also intimacy. In the medical terms, the upper and lower lips are known as labium superioris and labium inferioris respectively.^1^ Both the upper and lower lip consists of large amount of the mucous gland. Both the lips consist of the numerous amounts of grooves. The study of lip prints is called cheiloscopy.^2^

The fingerprints are widely used as an identifications aid. However, lip prints can be an additional tool for identification and during crime investigation.^3^ Studies from our settings can be valuable in providing data about the most common types of lip prints.

The aim of this study is to find out the prevalence of type I lip pattern among first- and second-year medical and dental students of a medical college.

## METHODS

The descriptive cross-sectional study was carried out in Kathmandu Medical College and Teaching Hospital in between November 2020 to January 2021 after getting the ethical clearance from the Institutional Review Committee (reference number- KMC-IRC 0311202006). All the medical and dental students of first and second years were included. Any history of surgery, trauma and ulcers on the lip were excluded. Participants were enrolled using convenient sampling and the sample size was calculated as:

$$\begin{array}{ll} \text{n} & ={\text{Z}}^{\text{2}}\times \text{p}\times \text{(1}-\text{p)}/{\text{e}}^{\text{2}}\\ & ={\left(1.96\right)}^{2}\times (0.50)\times (1-0.50)/{\left(0.04\right)}^{\text{2}}\\ & =601 \end{array}$$

The sample size was adjusted for finite population as,

$$\begin{array}{ll} {\text{n}}_{\text{o}} & =\text{(nN)}/[\text{N}+\text{(n}-\text{1)}]\\ & =(601\times 300)/[300+(601-1)\\ & =201 \end{array}$$

where,

n = minimum required sample size for infinite population
Z = 1.96 at 95% Confidence Interval (CI)
p = past prevalence taken as 50% for maximum sample size
e = margin of error, 4%
n_o_= adjusted sample size for finite population N= total number of first- and second-year medical and dental students= 300

Taking a 10% non-response rate, the sample size became 221. However, 240 participants were enrolled in the study.

The participations were included after taking verbal informed consent among the students in Kathmandu Medical College and Teaching Hospital between 18–20 years by using convenient sampling. The lips were wiped with the cotton properly inorder to remove the debris. And the dark red coloured lipstick was applied to the lips. Allow the lipstick immersed all over the vermillion borders of the lips. Then the cello tape was put over the lips. And the impression of lip in cello tape was pasted in the white A4 size paper. Then a lens was used to see the grooves of lip. The lips were divided into the four quadrants and the study was done respectively. The classification of patterns of the lines on the lips was done as proposed by TsuchihashiY.^5,6^

Type Ia: Clear-cut vertical groove that run across the entire lips.
Type Ib: Similar to type I, but do not cover the entire lip
Type II: Branched grooves (branching Y-shaped pattern)
Type III: Cross/intersected
Type IV: Reticular grooves
Type V-Undetermined

The data obtained was analysed with Statistical Package for the Social Sciences 20 version using descriptive statistics. Point estimate at 95% CI was calculated along with frequency and proportion for binary data.

## RESULTS

**Figure 1.**
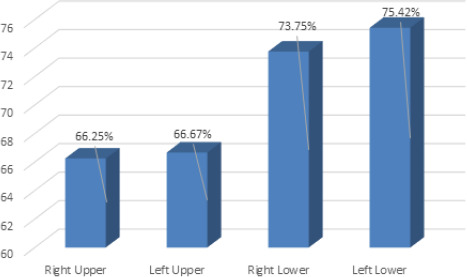
Distribution of Type I lip pattern among the four quadrants of the lip.

In this study, 190 (79.16%) (73.38-84.94 at 95% CI) students had type I print in at least one quadrant of their lips. Out of the four quadrants, left lower quadrant showed the maximum Type 1 in 181 (75.4%) (Figure 1).

One hundred and fifty-nine (66.3%) had type I pattern in the right upper quadrant (RUQ), 160 (66.7%) in left upper quadrant (LUQ), 177 (73.8%) in right lower quadrant (RLQ), and 181(75.4%) in left lower quadrant (LLQ). The maximum Type Ia was found in 115 (47.9%) and type Ib in 66 (27.5%) in at least one quadrant of the lip (Table 1).

**Table 1:** Distribution of Type I lip pattern among four quadrants of the lip.

Types	Right Upper Quadrant n (%)	Left Upper Quadrant n (%)	Right Lower Quadrant n (%)	Left Lower Quadrant n (%)
Type I	159 (66.3)	160 (66.7)	177 (73.8)	181 (75.4)
Type Ia	97 (40.4)	99 (41.3)	113 (47.1)	115 (47.9)
Type Ib	62 (25.8)	61 (25.4)	64 (26.7)	66 (27.5)

Our study showed that type I was the most prevalent lip pattern in both the genders. Type Ia was most common in females as 83 (34.5%) had type Ia in RUQ, 84 (35%) in LUQ, 79 (32.91%) in RLQ, and 79 (32.91%) in LLQ. Type Ib was more common in males as 45 (18.7%) had type Ib in RUQ, 45 (18.7%) in LUQ, 44 (18.3%) in RLQ, and 46 (19.16%) in LLQ respectively. Type V pattern was not found in our study. The second most common type was Type III in the upper lip in 67 (27.92%) and Type II in the lower lip in 60 (25%). Type IV was the least common pattern (Table 2).

**Table 2:** Gender wise distribution of lip patterns in each quadrant.

Lip pattern	Right Upper Quadrant		Left Upper Quadrant		Right Lower Quadrant		Left Lower Quadrant	
	Male	Female	Male	Female	Male	Female	Male	Female
	n (%)	n (%)	n (%)	n (%)	n (%)	n (%)	n (%)	n (%)
Type Ia	14 (5.8)	83 (34.5)	15 (6.25)	84 (35)	34 (14.16)	79 (32.91)	36 (15)	79 (32.91)
Type Ib	45 (18.75)	17 (7.08)	45 (18.75)	16 (6.6)	44 (18.33)	20 (8.3)	46 (19.16)	20 (8.3)
Type II	13 (5.4)	13 (5.41)	13 (5.4)	13 (5.41)	16 (6.6)	15 (6.25)	14 (5.83)	15 (6.25)
Type III	28 (11.6)	6 (2.5)	27 (11.25)	6 (2.5)	17 (7.08)	5 (2.08)	17 (7.08)	5 (2.08)
Type IV	20 (8.3)	1 (0.41)	20 (8.3)	1 (0.41)	9 (3.75)	1 (0.41)	7 (2.91)	1 (0.41)

**Figure 2.**
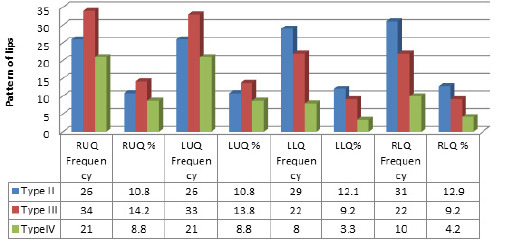
Distribution of the different types of lip pattern in all four Quadrants.

In this study, Type V was not determined in any quadrant of lip. The least types of lip pattern found in this study was Type IV in all the four quadrants of the lip. It was seen in 21 (8.8%) in the RUQ, 21 (8.8%) in the LUQ, 8 (3.5%) in the LLQ, and 10 (4.2%) in the RLQ (Figure 2).

## DISCUSSION

The lip prints are the permanent fold present in the entire life. In the world of forensic science there are various aid is used for personal identification. Nowadays, the lip prints are also used for aid of identification. During the crime investigation, the lip print can be visible or not in an object, If it is not visible lip prints can be developed by using various dyes and methods. The impressions can be collected and interpreted according to the above classification. ^5,6^

In this study, Type V was not found in the sample. In all the quadrants of the lip Type Ia was commonest and the Type Ib was the second one respectively. Whereas in the comparisons to other Types, Type IV was the least. Whereas in the four quadrants of lip, Type Ia was common in lower left and lower right respectively. In the entire quadrant Type IV was minimum. This finding was supported by S Gurung^7^ which was reported Type I was the common.

The commonest lip pattern found in this study was Type-Ia and Type-Ib. Type Ia was more prevalent in females and Type Ib more in males. This finding was in contrast to the study carried out in India, done by Aman Mital et al.^8^ which reported Type IV lip pattern was most prevalent pattern in males (44%) and Type I was more in female. Similar finding was seen in study conducted by Sharma et al.^9^ and Verghese et al.^10^ which showed type-IV as predominant type in male and Type 1 in female. The lip print pattern in male and female of Raipur district of Chattisgarh also showed Type Ia was most common in both male and female in which Type Ib was more common in male population and Type Ia was comparatively more in female.^10^ This variation in prevalence can be explained by the ethnic and racial differences of the several cohort studies.

This study was carried out in a limited sample size. The participants were enrolled using convenient sampling technique. Therefore, our results may not be generalizable to medical students all over Nepal. Further studies with a larger sample size and on a sample which is representative of medical students all over Nepal are required to know the actual prevalence.

## CONCLUSIONS

The lip print pattern Type I is the most common and the variation of lip print pattern is observed between the gender with Type Ia lip pattern more in female and Type Ib lip pattern more in male.
